# Paternal Diet-Induced Obesity Retards Early Mouse Embryo Development, Mitochondrial Activity and Pregnancy Health

**DOI:** 10.1371/journal.pone.0052304

**Published:** 2012-12-27

**Authors:** Natalie K. Binder, Natalie J. Hannan, David K. Gardner

**Affiliations:** Department of Zoology, University of Melbourne, Parkville, Victoria, Australia; Pennington Biomedical Research Center/LSU, United States of America

## Abstract

Worldwide, 48% of adult males are overweight or obese. An association between infertility and excessive body weight is now accepted, although focus remains primarily on females. It has been shown that parental obesity results in compromised embryo development, disproportionate changes in embryo metabolism and reduced blastocyst cell number. The aim of this study was to determine whether paternal obesity has negative effects on the resultant embryo. Specifically, using *in vitro* fertilisation (IVF), we wanted to isolate the functional effects of obesity on sperm by examining the subsequent embryo both pre- and post-implantation. Epididymal sperm was collected from age matched normal and obese C57BL/6 mice and cryopreserved for subsequent IVF with oocytes collected from Swiss females (normal diet/weight). Obesity was induced in male mice by feeding a high fat diet of 22% fat for 10 weeks. Resultant embryos were cultured individually and development monitored using time-lapse microscopy. Paternal obesity resulted in a significant delay in preimplantation embryo development as early as syngamy (P<0.05). Metabolic parameters were measured across key developmental stages, demonstrating significant reduction in mitochondrial membrane potential (P<0.01). Blastocysts were stained to determine trophectoderm (TE) and inner cell mass (ICM) cell numbers, revealing significant differences in the ratio of cell allocation to TE and ICM lineages (P<0.01). Functional studies examining blastocyst attachment, growth and implantation demonstrated that blastocysts derived from sperm of obese males displayed significantly reduced outgrowth on fibronectin *in vitro* (P<0.05) and retarded fetal development *in vivo* following embryo transfer (P<0.05). Taken together, these data clearly demonstrate that paternal obesity has significant negative effects on the embryo at a variety of key early developmental stages, resulting in delayed development, reduced placental size and smaller offspring.

## Introduction

Excessive body weight continues to be a significant problem plaguing modern society, and contrary to popular opinion, is not restricted to Western culture. Staggeringly, there are now more overweight people in the world than there are undernourished, estimated at 1.5 billion and 1 billion respectively [1]. The prevalence of overweight and obesity among the adult male population is exceedingly high, with countries such as USA, Nauru, Australia, Argentina, Greece and Samoa seeing rates increasing above 75% and 30% respectively [2]. Obesity is associated with numerous health problems, including cardiovascular disease, type 2 diabetes, hypertension, liver disease, psychosocial problems, cancer and infertility. Furthermore, the rate of obesity amongst men of reproductive age has tripled over the last three decades [3]. Although most research has focused on maternal obesity, there is growing data to indicate that paternal obesity is a cause for concern.

Previously we demonstrated that paternal obesity caused a delay in preimplantation embryo development from the second cleavage stage onwards [4]. This delay coincided with embryonic genome activation, suggesting paternal obesity is affecting sperm, possibly at the genetic or epigenetic level. A number of studies have investigated the effect of obesity on semen quality [5], [6], [7], [8], [9], [10], [11], [12], [13], [14], [15]. Recently, Fariello and colleagues reported decreased mitochondrial activity and progressive motility of spermatozoa and increased DNA fragmentation associated with obesity [16]. There is yet to be consensus as to the extent of the effect of obesity, if any, on semen quality, with numerous conflicting reports (for review [17]). Importantly, these studies have been conducted in humans and are thus subject to the confounding effect of differing lifestyles. Bakos and colleagues performed similar analysis in a mouse model of high fat diet and concluded obesity to be associated with decreased spermatozoa motility, capacitation and oocyte binding/fertilisation as well as increased intracellular reactive oxygen species and DNA damage [5].

Additionally, by using a mouse model of high fat diet, the effect of male obesity on embryo quality has been investigated. Paternal obesity has been shown to be associated with physiological changes to the preimplantation embryo, including significant delays in cell cycle progression, decreased blastocyst development and cell number, as well as increased glycolytic rate [4]. Mitchell and colleagues showed a significantly higher rate of 1-cell block in embryos from obese males and a decreased proportion of ‘on-time’ development from the 8-cell stage, culminating in reduced blastocyst cell number without changing the cell lineage allocation ratio [18]. Importantly, both of these studies generated embryos by mating animals. As such, the mechanism via which paternal obesity affects embryo quality cannot be defined, as several factors may have contributed including sperm, seminal fluid, time of mating and/or fertilisation, and behaviour/mounting ability. Following assisted reproduction in humans, paternal obesity has been associated with reduced blastocyst development and live birth rates [19]. However, this study was confounded by maternal weight, as obese males tend to have obese partners [20].

In the current study, we hypothesise that male obesity negatively affects sperm function, reducing its ability to generate a competent embryo capable of developing into a viable offspring. This would provide a mechanism for the creation of inferior embryos from obese males. As such, the aim of this study was to first generate an *in vitro* fertilisation (IVF) model of paternal obesity. This IVF model allowed us to control for the time of fertilisation, as well as removing other possible confounding factors such as changes to seminal fluid composition, animal behaviour and mating habits as a result of high fat feeding, which past models of paternal obesity have not been able to account for. The ability to fertilise oocytes, together with subsequent embryo physiology and fetal development were assessed as a measure of the functional effect of male obesity on sperm quality. Understanding this functional effect is paramount to developing strategies to minimise or rectify the effect of male obesity on not only reproductive fitness, but also the health of the ensuing generation.

## Materials and Methods

### Ethics Statement

This study was carried out in strict accordance with the *Australian code of practice for the care and use of animals for scientific purposes*. All protocols were approved by the Animal Ethics Committee of The University of Melbourne (Ethics Id: 1011628.1). All efforts were made to minimise suffering of animals.

### Experimental Animals, Diets, and Hormone Stimulation

Twenty 4 wk old male C57BL/6 mice were randomly assigned to one of two diets for 10 wks: control diet (4.8% fat, meat free rat and mouse chow), or high fat diet (22% fat [40% calories from fat], SF00-219; Specialty Feeds, Australia; http://www.specialtyfeeds.com/) manufactured to emulate a “Western-style fast food diet” (table S1). Mice on these diets were designated as normal and obese respectively. This high fat diet feeding regime does not affect fasting plasma glucose levels [5], [18], [21]. Animals were maintained in a 12 h light/dark photoperiod with food and water supplied *ad libitum*. Body weight was recorded weekly, and peritoneal fat deposits measured at euthanasia. Oocytes were collected from 4–6 wk old Swiss female mice fed control diet. Females were superovulated with intraperitoneal injections of 5 IU pregnant mare serum gonadotrophin (PMSG, Folligon, Intervet, UK) followed 48 h later by 5 IU human chorionic gonadotrophin (hCG, Chorulon, Intervet).

### Sperm Cryopreservation

Following the 10 wk feeding period, male mice were sacrificed for sperm collection and cryopreservation. Epididymal sperm was collected into cryoprotective medium of 18% raffinose (w/v), 3% skim milk (w/v) and 477 µM α-monothioglycerol [22]. All chemicals were obtained from Sigma-Aldrich (USA) unless otherwise stated. 10 µl aliquots of sperm were loaded into French straws and first cooled in liquid nitrogen vapours before being plunged into liquid nitrogen for storage.

### In Vitro Fertilisation

Cryopreserved sperm was thawed for 1 min in a 37°C water bath, to coincide with oocyte collection, at 14 h post-hCG. Sperm was then capacitated for 1 h in fertilisation medium (table S2 [23]) supplemented with 10 mg/ml human serum albumin (HSA; Vitrolife, Sweden). A Makler counting chamber was used to determine sperm concentration, where the number of spermatozoa counted in any strip of 10 squares indicates their concentration in millions per ml. Oocytes were collected by 15 h post-hCG in G-MOPS handling medium (table S2 [23]) supplemented with 5 mg/ml HSA, and transferred to 40 µl drops of fertilisation medium under paraffin oil (Ovoil, Vitrolife) in 6% CO_2_, 5% O_2_ and 89% N_2_ at 37°C. 1–2 million motile sperm/ml were added to 1–2 cumulus oocyte complexes, and fertilisation allowed to occur over a 4 h window. Following fertilisation, pronucleate oocytes were washed once in both fertilisation medium and G1 medium (table S2 [24]) prior to culture.

### Embryo Culture and the Analysis of Cleavage Rate and Cell Cycle Times

Embryos were cultured individually in 2 µl drops of G1 medium supplemented with 5 mg/ml HSA under paraffin oil in 6% CO_2_, 5% O_2_ and 89% N_2_ at 37°C in a humidified multi-gas imaging incubator (Sanyo MCOK-5M[RC], Japan) for 48 h. Embryos were then transferred to G2 medium supplemented with 5 mg/ml HSA (table S2 [24]) for a further 44 h. Time-lapse images of individual embryos were generated every 15 min throughout culture. The timing of syngamy, the first, second and third cleavage events, as well as the start of cavitation and hatching were calculated in hours post-fertilisation, and cell cycle length determined as previously established [25].

### Mitochondrial Membrane Potential

The mitochondrial probe JC-1 (5,5′,6,6′-tetrachloro-1,1′,3,3′-tetraethylbenzimidazolyl-carbocynanine iodide; Molecular Probes, USA) was used to determine mitochondrial membrane potential. JC-1 accumulates in mitochondria in a membrane potential-dependent manner, indicated by fluorescence emission shift from green (∼ 529 nm) to red (∼ 590). Decreasing red : green fluorescence intensity indicates mitochondrial depolarisation. Four-cell embryos were stained with JC-1 (1.5 mM) for 15 min at 37°C in the dark. Embryos were washed in loading media (GMOPS, no protein) and loaded onto a glass slide under a coverslip, and imaged immediately on a heated stage using epifluorescence (Nikon Eclipse Ti, Japan). Images were analysed using the accompanying NIS-elements software package. The average red and green pixel intensity was determined and mitochondrial membrane potential expressed as the ratio of red intensity (high membrane potential) to green intensity (low membrane potential).

### Assessment of Pyruvate and Glucose Consumption, and Lactate Production

At 36 h post-fertilisation, pyruvate uptake by 4-cell embryos was assessed immediately prior to JC-1 staining. At 94 h post-fertilisation, glucose uptake and lactate production by blastocysts was assessed prior to either differential cell stain or embryo outgrowth. Individual embryos were placed into 50 nl drops of incubation medium at 37°C for analysis. Incubation medium for 4-cell stage embryos was G-MOPS+HSA containing 0.5 mM glucose, 10 mM lactate and 0.35 mM pyruvate. Incubation medium for blastocyst-stage embryos was G-MOPS+HSA modified to contain 0.5 mM glucose as the sole energy source. A 1 nl sample of media was taken per embryo per metabolite analysed. Levels of pyruvate or glucose and lactate in the media were assessed using ultramicrofluorimetry as previously described [26], [27]. Glycolytic activity of the blastocysts was calculated on the basis that 2 mole of lactate is produced per 1 mole of glucose consumed by the embryo [28].

### Differential Cell Staining

Allocation of cells to the trophectoderm (TE) and inner cell mass (ICM) was assessed in blastocyst-stage embryos following final morphological assessment 94 h post-fertilisation, by differential staining [29]. Blastocysts were incubated in 0.5% pronase to remove the zona pellucida before being washed in G-MOPS (performed at 37°C unless otherwise stated). Blastocysts were then incubated in 10 mM TNBS (2,4,6-trinitrobenzene sulfonic acid) in the dark at 4°C for 10 min and washed again, before incubation in 0.1 mg/ml anti-DNP BSA (anti-Dinitrophenol bovine serum albumin) for 10 min. Following this, blastocysts were washed and incubated in 10% guinea pig serum with 25 mg/ml propidium iodide in the dark for 8 min. Blastocysts were immediately transferred to a solution of 0.1 mg/ml Bisbenzimide (Hoescht, 33342) in 10% ethanol for 15 min and then washed in G-MOPS. Propidium iodide is unable to penetrate tight gap junctions between cells to reach the ICM and as such stains only TE cells red (∼ 620 nm). Bisbenzimide stains all cells of the blastocyst blue (∼ 470 nm). Blastocysts were subsequently mounted in glycerol under cover slips and cell number counted under fluorescent light (Nikon TS100-F), with cells of the TE appearing pink and ICM appearing blue.

### Embryo Outgrowths

At 98 h post fertilisation, embryo outgrowths were performed [30]. Flat-bottomed 96-well tissue culture dishes were rinsed with sterile PBS and coated with a solution of 10 µg/ml fibronectin (BD Biosciences, USA). Coated wells were rinsed with sterile PBS and incubated with a 4 mg/ml BSA PBS solution for 2 h. Wells were subsequently rinsed with PBS, followed by G2 medium, and then filled with 150 µl of G2 medium, supplemented with 5% fetal calf serum, and equilibrated at 37°C under paraffin oil (Ovoil) under the same gas-phase conditions as embryo culture for 4 h before the addition of blastocysts. Expanded and hatching blastocysts were placed individually into the coated wells (one embryo per well) and incubated for 72 h. Outgrowth was examined and images were taken at a matching magnification (×10) at 48, 66 and 72 h after transfer to outgrowth plate with an inverted microscope (Nikon Eclipse Ti) equipped with a heated stage at 37°C. The extent of outgrowth for each treatment was obtained by measuring the area of outgrowth in each of the images taken across the experiment using NIS Elements BR 3.00, SP7 Laboratory Imaging software (Nikon). All images were analysed at matching magnification.

### Embryo Transfer

F1 (C57BL/6×CBA/Ca) female mice between 8 and 12 wks of age were mated with vasectomised males to induce pseudopregnancy. Mating was confirmed by the presence of a vaginal plug. Embryos were then transferred on day 4 of development (asynchronous to recipient female reproductive tract, which was staged at day 3.5 of pregnancy). Recipient female mice were anesthetised with an intraperitoneal injection of ketamine (75 mg/kg Ketalar, Pfizer, Australia) and medetomidate (1 mg/kg Domitor, Pfizer). Five embryos were transferred through a small dorsal incision, with a glass pipette, into the lumen of each uterine horn. Each recipient female received embryos from both paternal groups (normal and obese). Alternate groups were transferred to both the right and left horn per recipient, to avoid any preferential implantation bias of the left or right horn. There is no transuterine migration of blastocysts after embryo transfer in mice [31]. Following embryo transfer, the skin wound was sealed with sterile surgical clips, and the recipient female underwent postoperative recovery with an intraperitoneal injection of atipamezole (1 mg/kg Antisedan, Pfizer) to reverse the effects of medetomidate, while the analgesic effects of ketamine remained. Pregnant females were sacrificed 10 days later (pseudopregnant day 13.5). The number of fetuses and implantation absorption sites was recorded to determine rates of implantation and fetus development. Fetal and placental weight was recorded, and crown-rump length measured. Fetal ear, eye and limb development was compared [32], [33]. Morphological grades for each feature – ear, eye, limb – of 12.5, 13, 13.5, 14 and 14.5 correspond to having the same morphological characteristics of *in vivo* derived fetuses on embryonic days 12.5, 13, 13.5, 14 and 14.5 respectively, as devised by Wahlsten and Wainwright [33].

### Statistical Analysis

Distribution of data was assessed with D’Agostino and Pearson omnibus normality test, results of which determined if the data distribution was ‘normal’ and whether parametric or non-parametric analysis was conducted. All statistical analysis was carried out using PRISM version 3.00 for Windows (GraphPad, San Diego, CA).

#### Weight gain and fat deposits

Initial body weight, final body weight, total weight gained and peritoneal fat mass (g) as well as fat mass normalised to body weight was compared between control fed (normal) and high fat fed (obese) age-matched male mice. Data was normally distributed and statistically analysed with a paired t-test (parametric).

#### Fertilisation rate

The rate of fertilisation from each round of IVF (n = 26 rounds) was compared between the normal and obese groups. Data was normally distributed and statistically analysed with a paired t-test (parametric).

#### Time-lapse (embryo developmental kinetics)

The time, in hours (h) from fertilisation (occurring at 18 h post-hCG), to each clearly definable developmental stage (syngamy, 2-cell, 3-cell, 4-cell, 5-cell, 6-cell, 7-cell, 8-cell, cavitation, hatching) as well as the time between each of these developmental stages were not normally distributed and statistically analysed with a Mann-Whitney test (non-parametric).

#### Metabolism

Pyruvate uptake by 4-cell embryos and glucose uptake and lactate output by blastocysts (pmol/embryo/h) were not normally distributed and statistically analysed with a Mann-Whitney test (non-parametric). The ratio of lactate output to glucose uptake (glycolysis) and red : green fluorescence intensity of JC-1 probed 4-cell embryos (mitochondrial membrane potential) were also not normally distributed and statistically analysed with a Mann-Whitney test (non-parametric).

#### Cell count

The number of cells counted in the ICM and TE (combined to give total cell number), as well as the ratio of ICM to TE cells was compared between normal and obese groups. Data were not normally distributed and statistically analysed with a Mann-Whitney test (non-parametric).

#### Blastocyst outgrowths

The rate of blastocyst attachment to fibronectin in culture (%) was compared between the normal and obese groups with a contingency table and Fisher’s exact test. The area outgrown by blastocysts successfully attached (pixel^2^) was not normally distributed and statistically analysed with a Mann-Whitney test (non-parametric).

#### Embryo transfer

The proportion of embryos implanted per transfer, developed per transfer and developed per implantation were compared between the normal and obese groups with a contingency table and Fisher’s exact test. Fetal and placental weight (g), crown-rump length (mm) and fetal morphological grade were not normally distributed and statistically analysed with a Mann-Whitney test (non-parametric).

## Results

### Dietary Effects on Weight Gain and Fat Deposits

**Figure 1:**
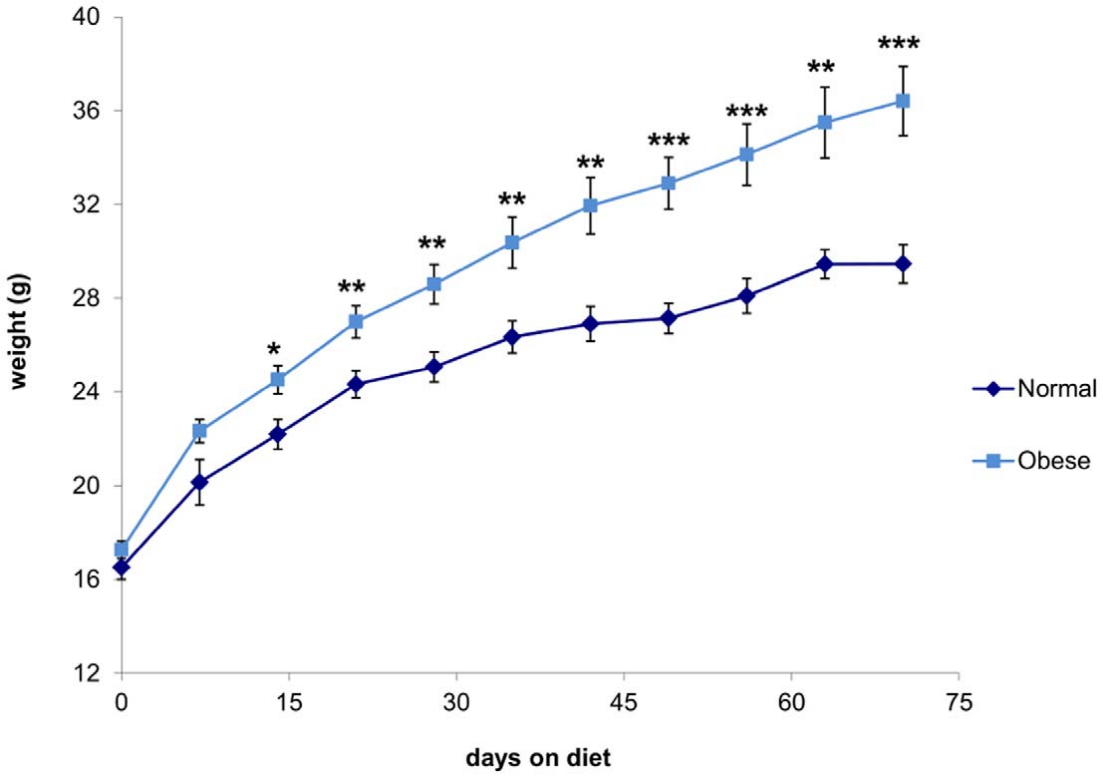
Male C57BL/6 mice fed a high fat diet over 10 wks demonstrated significant weight gain. The dark line represents mice fed control diet (designated normal) and the light line represents mice fed high fat diet (designated obese). Data are expressed as mean ± SEM. * P<0.05, ** P<0.01, *** P<0.001, n = 10 male mice per diet.

Male C57BL/6 mice fed the high fat diet for 10 wks (obese) gained significantly more weight than mice fed the control diet (normal), to weigh significantly more at the time of sperm collection (figure 1). Obese mice also had significantly increased peritoneal fat deposits compared to normal mice, which did not normalise with weight (table 1). Such changes to body composition with high fat feeding in this mouse strain have been previously reported [4].

**Table 1:** Average body weight, weight change and body fat of normal and obese male C57BL/6 mice following 10 wk feeding period.

Parameter	Normal	Obese	P value
Initial weight (g)	16.51±0.51	17.26±0.37	NS
Final weight (g)	29.46±0.82	36.41±1.48	<0.01
Weight gain (g)	12.82±0.82	19.93±1.51	<0.01
Peritoneal fat (g)	1.00±0.16	2.58±0.16	<0.001
Proportion fat (% BW)	2.83±0.36	6.01±0.24	<0.001

Data are expressed as mean ± SEM. n = 10 male mice per diet; g = gram; BW = body weight; NS = not significant.

### In Vitro Fertilisation (IVF)

Sperm from obese males fertilised oocytes at a significantly higher rate than sperm from normal males (41.82±3.57% vs. 31.22±3.01% respectively; P<0.05).

### Obesity Effects on Embryo Developmental Kinetics

Embryos generated from the sperm of obese males showed significant delays in preimplantation development from as early as syngamy (table 2). The initial delay occurring at syngamy of approximately 15 min was extended at cleavage to the 3-cell stage to almost 1 h compared to normal counterparts. The kinetic delays observed are the result of two separate cell cycle extensions in the precompaction development of these embryos. Embryos derived from the sperm of obese males had a slower progression from syngamy to 2-cell cleavage than normal embryos (1.83±0.2 h vs. 1.74±0.02 h respectively; P<0.01), as well as a longer cell cycle length for cleavage from 2- to 3-cell (21.74±0.14 h vs. 21.13±0.11 h respectively, P<0.01). The retardation in developmental kinetics was maintained without further exacerbation for the precompaction period. Postcompaction events (time of cavitation and hatching) were not affected by paternal obesity, however the frequency of blastocyst formation was significantly reduced compared to embryos derived from the sperm of normal males at 94 h post fertilisation (41.03% vs. 58.92% respectively; P<0.001).

**Table 2:** Timing of syngamy, 1^st^, 2^nd^, 3^rd^ cleavage stages, cavity formation and initiation of hatching for embryos derived from the sperm of normal and obese males.

Developmental event		Normal	Obese	P value
	Cleavage *(hours post-fertilisation)*			
Syngamy		12.63±0.09	12.86±0.07	<0.05
1^st^ cleavage	*2-cell*	14.35±0.08	14.66±0.07	<0.001
2^nd^ cleavage	*3-cell*	35.51±0.14	36.35±0.15	<0.0001
	*4-cell*	36.45±0.16	37.34±0.17	<0.001
3^rd^ cleavage	*5-cell*	45.99±0.17	46.92±0.19	<0.001
	*6-cell*	46.60±0.18	47.49±0.19	<0.01
	*7-cell*	47.33±0.19	48.16±0.20	<0.01
	*8-cell*	47.96±0.20	48.80±0.23	<0.05
Cavitation		76.47±0.44	77.24±0.43	NS
Hatching		90.88±0.76	91.02±0.85	NS

Data are expressed as mean ± SEM. n = 200 embryos each per paternal group; NS = not significant.

### Obesity Effects on Mitochondrial Membrane Potential

**Figure 2:**
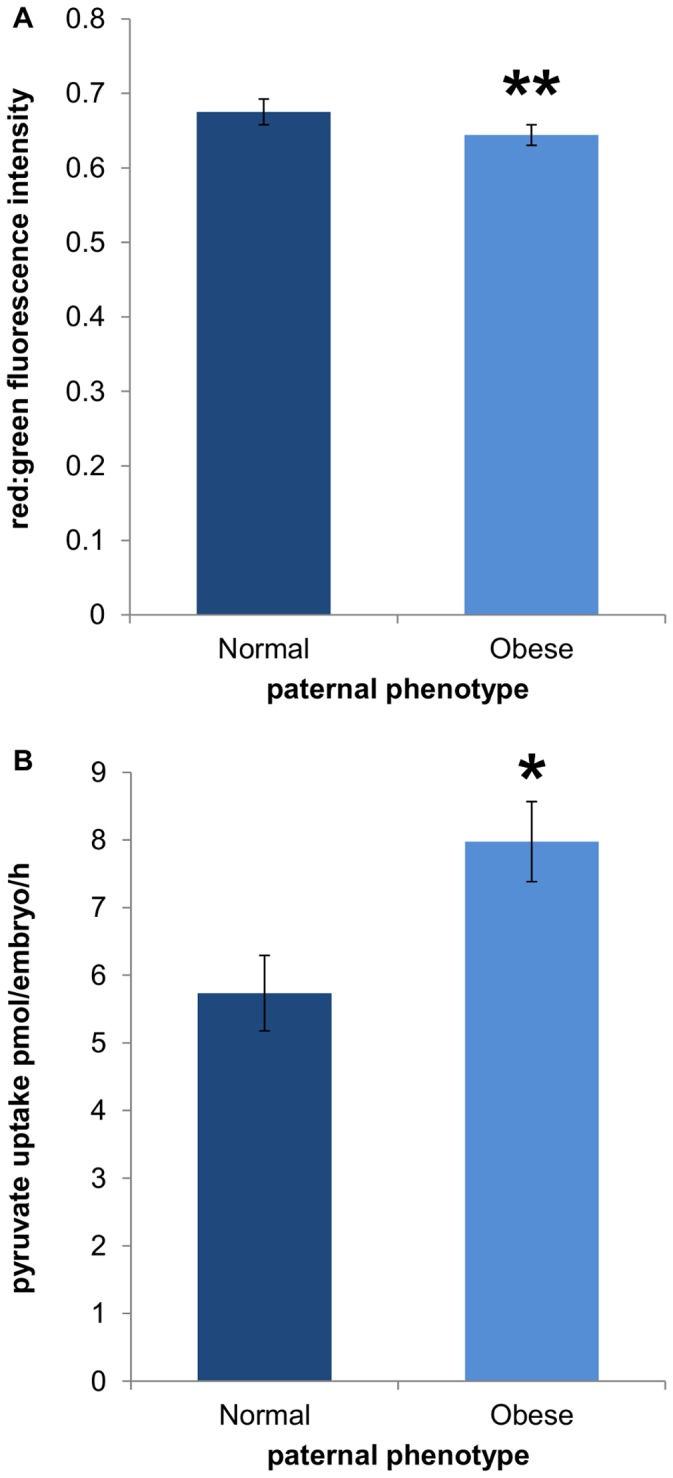
Embryos derived from obese males had significantly decreased mitochondrial membrane potential and increased pyruvate uptake. Dark bars represent embryos derived from normal male sperm and light bars represent embryos derived from obese male sperm for both mitochondrial membrane potential (A) and pyruvate uptake (B), at the 4-cell stage. Data are expressed as mean ± SEM. * P<0.05, ** P<0.01, n = 26 embryos per paternal group.

A significant reduction in the red : green fluorescence intensity of the JC-1 probe was observed in 4-cell embryos derived from the sperm of obese males compared to normal (figure 2A). This difference indicates a reduced mitochondrial membrane potential of 4-cell embryos derived from obese males. Measured concurrently, pyruvate uptake of these 4-cell embryos was significantly increased (figure 2B).

### Obesity Effects on Embryo Metabolism

Neither glucose consumption (normal 9.31±0.40 pmol/embryo/h vs. obese 8.63±0.30 pmol/embryo/h; P = 0.17) nor lactate production (normal 6.70±0.40 pmol/embryo/h vs. obese 7.06±0.33 pmol/embryo/h; P = 0.47) varied significantly between embryos generated from the sperm of normal or obese males. Normalising for cell number did not alter this outcome, as blastocysts had equivalent cell number at the time of analysis. As such, the glycolytic rate was the same for embryos from both normal and obese paternal groups (36.93±1.89% vs. 42.10±1.93% respectively; P = 0.08).

### Obesity Effects on Cell Lineage Allocation in the Blastocyst

**Figure 3:**
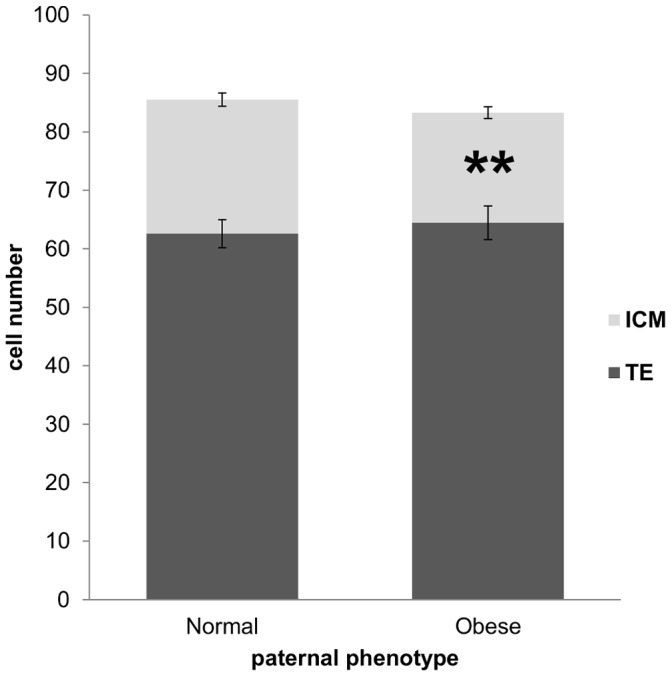
ICM:TE was significantly decreased in embryos from obese males while total cell number was unchanged. The dark portion of the bars represents the average number of trophectoderm cells and the light portion the average number of inner cell mass cells. Data are expressed as mean ± SEM. ** P<0.01, n = 60 embryos per paternal group.

Blastocysts from both normal and obese paternal groups had the same total cell number (85.56±2.82 vs. 83.11±3.44 respectively; P = 0.88) and allocation of cells to the TE (62.60±2.38 vs. 64.45±2.86 respectively; P = 0.47). However, compared to normal, embryos from the obese paternal group had significantly reduced allocation of cells to the ICM (22.93±1.14 vs. 18.82±1.00 respectively; P<0.01; figure 3), resulting in a decreased ICM : TE ratio (0.40±0.03 vs. 0.31±0.02 respectively; P<0.01).

### Obesity Effects on Blastocyst Outgrowth

**Figure 4:**
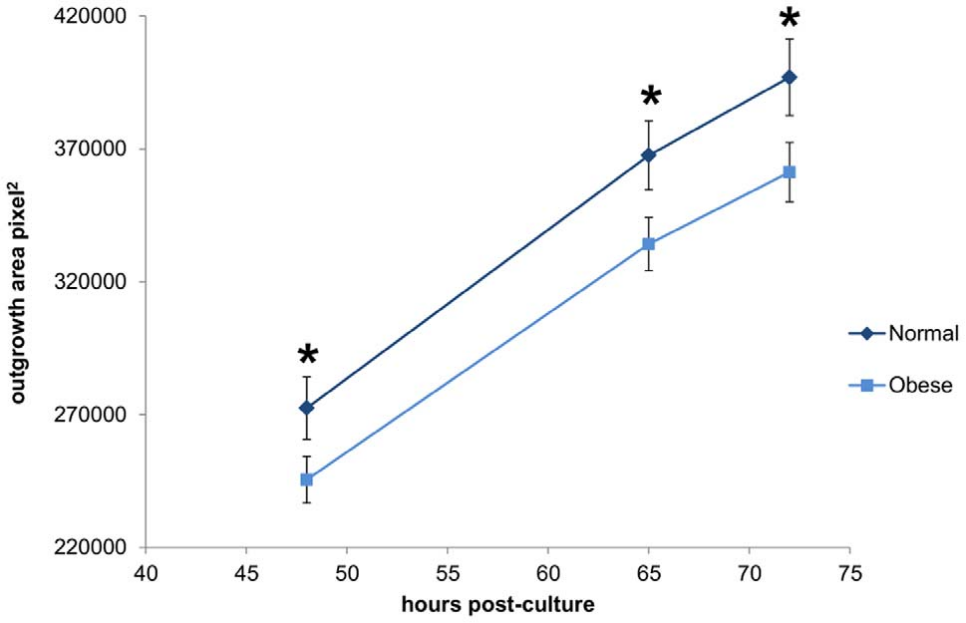
Blastocyst outgrowth was significantly reduced in embryos derived from the sperm of obese males. The dark line represents embryos derived from normal male sperm and the light line represents embryos derived from obese male sperm. Data are expressed as mean ± SEM. * P<0.05, n = 44 embryos outgrown per paternal group.

Blastocysts from the normal and obese paternal groups attached to fibronectin in culture at the same rate (75.48±9.56% vs. 78.33±6.54% respectively; P = 0.82). However, paternal obesity was associated with a significant reduction in the ability of the blastocyst to outgrow on fibronectin (figure 4).

### Obesity Effects on Pregnancy

**Figure 5:**
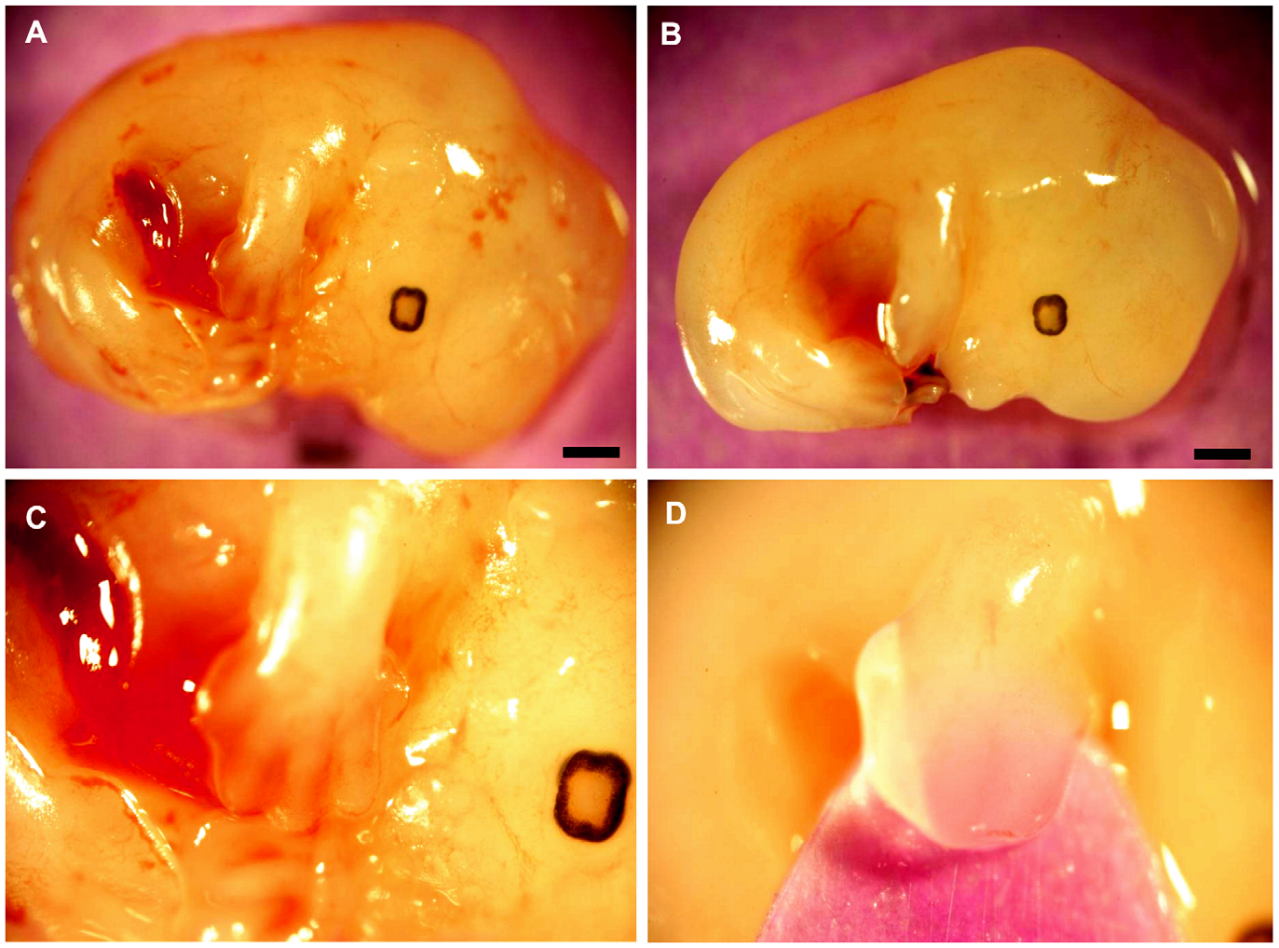
Retarded fetal morphological development was observed in embryos derived from the sperm of obese males. Typical fetal morphology on pseudopregnancy day 13.5 of fetuses generated from normal sperm **(**A). Delayed morphological characteristics and size in fetuses generated from the sperm of obese males (B) is clearly observed. Higher power (×4 magnification) images of forelimb development in fetuses derived from normal (C) and obese (D) male sperm. Depicting defined digits in the normal (C) and delayed development of the forelimb and digits in fetuses derived from obese male sperm (D). Scale bar = 1 mm.

Embryos derived from the sperm of obese males had a reduced ability to implant in the uterine wall of a recipient female following blastocyst transfer (table 3). The rate of ongoing pregnancy was also reduced for embryos derived from the sperm of obese males. However, when normalised to implantation rate, ongoing pregnancy did not differ from normal (table 3). Both fetal and placental weights were significantly reduced in conceptuses of obese males compared to normal. Furthermore, fetal development was markedly altered with paternal obesity resulting in retarded limb morphology, and decreased crown-rump length (figure 5). Fetuses from the obese paternal group typically had smooth footplates (figure 5D) compared to those from the normal paternal group, which at day 13.5 of pseudopregnancy, had clearly defined, webbed digits (figure 5C). With morphological retardation, as well as shortened crown-rump length and reduced fetal and placental weight, fetuses from the obese paternal group show a delay in development of approximately 0.25 days compared to normal, using the morphological grading scheme defined by Wahlsten and Wainwright for mouse fetal development [33].

**Table 3:** Pregnancy outcomes from transfer of embryos generated from the sperm of normal and obese males into pseudopregnant recipient female mice.

Parameter	Normal	Obese	P value
Implantation/ET (%)	79.00	67.29	<0.01
Ongoing pregnancy/ET (%)	54.00	43.93	<0.05
Ongoing pregnancy/implantation (%)	68.35	65.28	NS
Fetal weight (mg)	131.91±3.38	122.16±3.30	<0.05
Placental weight (mg)	139.67±4.48	125.49±4.44	<0.05
Fetal/placental ratio	0.98±0.03	1.02±0.04	NS
Crown-rump length (mm)	10.25±0.11	9.86±0.11	<0.01
Limb morphological grade	13.70±0.06	13.36±0.08	<0.001
Eye morphological grade	14.03±0.04	13.94±0.07	NS
Ear morphological grade	13.17±0.06	13.02±0.07	NS

Data are expressed as mean ± SEM, except for percentage date. Fetal morphological grade was determined using the system adapted from Wahlsten and Wainwright [33]. n = 100 embryos transferred per paternal group; mg = milligram; mm = millimetre; ET = embryo transferred; NS = not significant.

## Discussion

While the negative impact of obesity on embryo quality has recently been shown [4], [18], [19], the underlying mechanisms are yet to be elucidated. Furthermore, the extent of the effect, if any, of obesity on semen parameters remains inconclusive. The data from this study demonstrate for the first time that obesity negatively affects the functional capacity of sperm to generate a high quality embryo capable of achieving a healthy pregnancy. Incorporating *in vitro* fertilisation into a model of paternal diet-induced obesity diminished the effect of confounding factors such as changes to seminal fluid composition, time of fertilisation and mating behaviours. Fertilising oocytes with the sperm of obese males resulted in significant changes in resultant embryo mitochondrial activity and kinetic development. While changes were permissive to blastocyst formation – albeit at a reduced frequency – cell lineage allocation varied significantly from normal, and fetal and placental development was compromised.

In this study, sperm from obese males fertilised oocytes at a higher rate than sperm from normal males. Previously, Bakos and colleagues found male obesity to be negatively correlated with both oocyte binding and fertilisation [5]. Interestingly, the previous study was performed with fresh sperm whereas the current study used cryopreserved sperm, suggesting that sperm from obese males may have improved cryotolerance compared to normal. The membrane fluidity of spermatozoa has been shown to play a vital role in response to cryopreservation, and varies markedly between individuals. Higher membrane fluidity confers better cryotolerance [34]. Membrane fluidity is modulated by the cholesterol to phospholipid ratio, and high levels of membrane cholesterol have been shown to maintain membrane fluidity across a range of temperatures [35]. High fat feeding similar to the diet used in this study results in increased levels of circulating cholesterol, free fatty acids and triglycerides [18], [21]. High cholesterol feeding in rabbits affects cholesterol distribution in spermatozoa, suggesting that circulating lipids are able to enter the epididymal lumen [36]. As such, it is possible that in this study, high fat feeding has resulted in changes to spermatozoa membrane dynamics due to increased circulating cholesterol levels, altering membrane fluidity, and as such improving cryotolerance compared to normal. Further studies are required to investigate this finding.

Our model of *in vitro* fertilisation combined with time-lapse microscopy has shown for the first time that paternal obesity affects embryo developmental kinetics as early as syngamy, causing approximately 15 min delay in pronuclear fusion. This relatively small difference in timing could be detected because of our tightly controlled timing of fertilisation and use of high temporal time-lapse microscopy. Unlike other mammalian species, mouse spermatozoa do not contribute centriole to the fertilised oocyte, and as such there is no paternal involvement in the organisation of the aster that draws the male and female pronuclei together. Instead, we would suggest that this delay in syngamy could be due to changes in chromatin decondensation as the polyamines surrounding the male pronuclear DNA are replaced with oocyte histones. This observed delay in kinetic development was exacerbated at the second cleavage event to approximately 1 h, as a result of an extension of the cell cycle length from the 2- to 3-cell stage, coinciding with embryonic genome activation. This reinforces our previous findings and provides further support for a genetic effect of paternal obesity on reproductive fitness [4].

Shortly after activation of the embryonic genome, 4-cell stage embryos were assessed for mitochondrial function. The generation of a mitochondrial membrane potential (MMP) drives energy production by enabling oxidative phosphorylation of pyruvate, and has been used to reflect variations in mitochondrial function in the embryo [37], [38], [39]. Embryos derived from the sperm of obese males had significantly decreased MMP compared to normal. Despite mitochondrial biogenesis being absent during the preimplantation period, mitochondrial DNA is actively transcribed from the 2-cell stage to produce respiratory chain subunits [40], of which paternal nuclear DNA encoded proteins would be incorporated. Measured concurrently, pyruvate uptake was significantly increased in embryos derived from the sperm of obese males compared to normal. This increase in pyruvate uptake could be a compensatory mechanism by the embryo to allow for the decreased MMP, or conversely, MMP may have been down regulated to tolerate the higher than normal influx of pyruvate. Alternatively, pyruvate acts as a strong antioxidant [41], [42], [43], [44] and increased uptake may be to combat oxidative stress that is affecting MMP. Interestingly, by the blastocyst stage, metabolism of embryos derived from the sperm of obese males was the same as normal embryos, with both groups having glycolytic rates close to 40%, considered within the optimal range for successful implantation [28] and comparable to *in vivo* blastocysts [27]. It has previously been shown that blastocysts derived from obese males have a significantly higher glycolytic rate than normal [4]. However, these blastocysts were generated by mating, and as such, other factors are likely involved.

The overall rate of blastocyst formation reported here appears somewhat low. It is important to note however the relatively short *in vitro* culture period of 94 h, after which no further morphological assessment was performed. Furthermore, the lower rate of development was not surprising given the background line (C57BL/6) and the stress of IVF. The frequency of blastocyst development was significantly reduced with paternal obesity, despite cavitation and hatching occurring normally. The blastocysts that did form however, had similar cell numbers to normal. Further investigation revealed paternal obesity was associated with a significant reduction in allocation of cells to the ICM compared to the TE. This is the first study to find a difference in cell lineage allocation associated with paternal obesity, and may again reflect a difference with this model examining the functional effect of obesity on sperm, as opposed to the general effect of paternal obesity on embryo quality. The reduction in ICM cell number here suggests there may be consequences to the fetus. Also, the volume of ICM has been associated with the degree of trophoblast proliferation following outgrowth or transfer and it is thought that the ICM may have a governing role during implantation [45]. Changes in gene imprint status – evident through experiments involving androgenotes and parthogenotes in which either embryonic or extra-embryonic tissue is compromised respectively (for review [46]) – could potentially elicit such changes in cell allocation.

Given this, blastocyst outgrowths and embryo transfers were performed to determine whether there was a functional effect of this reduction in ICM. Embryos derived from the sperm of obese males had a significantly reduced ability to outgrow over 72 hours of culture, consistent with the suggestion that a decreased ICM will correlate with reduced outgrowth capacity. Morphologically similar blastocysts from both paternal groups were transferred to pseudopregnant recipient females. Similar to the findings of Mitchell and colleagues [18], paternal obesity was associated with a significant reduction in both implantation and ongoing pregnancy rates. However, the current study also demonstrates a significant reduction in both fetal and placental weight, crown-rump length and limb morphology with paternal obesity. Reduced fetal weight could be due directly to reduced ICM, or reflect changes in placental function [47], [48]. In a model of increasing mitochondrial inhibition [49], mitochondrial dysfunction significantly affects normal embryonic, fetal and placental development. As such, it could be suggested that the decreased mitochondrial activity shown at the 4-cell stage is a key contributor to the adverse pregnancy outcomes associated with paternal diet-induced obesity.

Identified for the first time in this study, reduced placental and fetal growth associated with paternal obesity is in contrast to the effect of maternal obesity on pregnancy, which correlates significantly with the incidence of large for gestational age births [50], [51]. Reduced placental growth generally precedes reduced fetal growth [52], [53], and placenta size has been identified as an independent determinant of both intrauterine fetal growth and birth weight [54]. Paternally expressed imprinted genes tend to enhance placental growth [55]. As such, imprinting errors or epigenetic changes originating during spermatogenesis could contribute to the reduction in placental and consequent fetal growth associated with paternal obesity. Reduced intrauterine growth is a well established risk factor for adverse long term health, increasing the likelihood of coronary heart disease, type 2 diabetes, stroke and hypertension in adulthood, independent of lifestyle (for review [56]). Reduced fetal growth can also alter the body’s response to nutrition later in life. Small for gestational age offspring have reduced hypothalamic neural satiety pathways leading to programmed overeating – creating a self perpetuating cycle of obesity [57], [58].

In conclusion, this is the first study to combine *in vitro* fertilisation (IVF) with male diet-induced obesity to investigate the functional effect of male obesity on embryo development and pregnancy. The current study has clearly demonstrated that there are several consequences of male obesity on embryo development, implantation, pregnancy success and fetal size. Changes to sperm development, seminal fluid composition and animal mating behaviour with high fat feeding likely impact reproductive success further and requires additional investigation. Additionally, studies employing side-by-side natural mating and *in vitro* culture would help to elucidate any confounding effects of sperm cryopreservation, IVF and culture conditions.

## Supporting Information

Table S1 **Nutritional content of control and high fat diets feed to mice for 10 wks to generate normal and obese male mice respectively.** Data as reported by manufacturer; Specialty Feeds, Australia; http://www.specialtyfeeds.com/.
(DOCX)

Table S2 **Composition of handling, IVF and sequential embryo culture media.** G-MOPS and Fertilisation media adapted from [22]; G1 and G2 sequential culture media adapted from [23].
(DOCX)
