# Supplementary material for: Paternal Diet-Induced Obesity Retards Early Mouse Embryo Development, Mitochondrial Activity and Pregnancy Health
Source: PLoS One. 2012 Dec 27;7(12):e52304. doi: 10.1371/journal.pone.0052304 (PMC3531483; doi:10.1371/journal.pone.0052304)
Supplement: Table S1 — Nutritional content of control and high fat diets feed to mice for 10 wks to generate normal and obese male mice respectively. Data as reported by manufacturer; Specialty Feeds, Australia; http://www.specialtyfeeds.com/. (DOCX) [file pone.0052304.s001.docx]

**Table S1. Nutritional content of control and high fat diets feed to mice for 10 wks to generate normal and obese male mice respectively.**

|  |  | **Control diet** | **High fat diet** |
| --- | --- | --- | --- |
| Amino acids | Valine | 0.87% | 1.20% |
|  | Leucine | 1.40% | 1.80% |
|  | Isoleucine | 0.80% | 0.80% |
|  | Threonine | 0.70% | 0.80% |
|  | Methionine | 0.30% | 0.80% |
|  | Cystine | 0.90% | 0.06% |
|  | Lysine | 0.90% | 1.50% |
|  | Phenylanine | 0.90% | 1.00% |
|  | Tyrosine | 0.50% | 1.00% |
|  | Tryptophan | 0.20% | 0.30% |
|  | Histidine | 0.53% | ‒ |
| Minerals | Calcium | 0.80% | 0.60% |
|  | Phosphorous | 0.70% | 0.30% |
|  | Magnesium | 0.20% | 0.10% |
|  | Sodium | 0.20% | 0.12% |
|  | Chloride | ‒ | 0.16% |
|  | Potassium | 0.70% | 0.40% |
|  | Sulphur | 0.20% | 0.23% |
|  | Iron | 590mg/kg | 80 mg/kg |
|  | Copper | 24 mg/kg | 7.0 mg/kg |
|  | Iodine | 0.5 mg/kg | 0.2 mg/kg |
|  | Manganese | 115 mg/kg | 20 mg/kg |
|  | Cobalt | 1.0 mg/kg | No data |
|  | Zinc | 90 mg/kg | 52 mg/kg |
|  | Molybdenum | 1.2 mg/kg | 0.15mg/kg |
|  | Selenium | 0.4 mg/kg | 0.3 mg/kg |
|  | Cadmium | 0.04 mg/kg | No data |
|  | Chromium | ‒ | 1.0 mg/kg |
|  | Fluoride | ‒ | 1.0mg/kg |
|  | Lithium | ‒ | 0.1 mg/kg |
|  | Boron | ‒ | 2.3 mg/kg |
|  | Nickel | ‒ | 0.5 mg/kg |
|  | Vanadium | ‒ | 0.1 mg/kg |
| Vitamins | Vitamin A (retinol) | 19500 IU/kg | 11650 IU/kg |
|  | Vitamin D (cholecalciferol) | 2000 IU/kg | 1100 IU/kg |
|  | Vitamin E (a tocopherol acetate) | 110 mg/kg | 64 mg/kg |
|  | Vitamin K (menadione) | 20 mg/kg | 12.5 mg/kg |
|  | Vitamin C (ascorbic acid) | No data | 700 mg/kg |
|  | Vitamin B1 (thiamine) | 80 mg/kg | 11 mg/kg |
|  | Vitamin B2 (riboflavin) | 30 mg/kg | 11 mg/kg |
|  | Niacin (nicotinic acid) | 145 mg/kg | 50 mg/kg |
|  | Vitamin B6 (pyridoxine) | 28 mg/kg | 11 mg/kg |
|  | Pantothenic acid | 60 mg/kg | 34 mg/kg |
|  | Biotin | 410 mg/kg | 200 µg/kg |
|  | Folic acid | 5 mg/kg | 1 mg/kg |
|  | Inositol | No data | 55 mg/kg |
|  | Vitamin B12 (cyancobalamin) | 150 µg/kg | 18 µg/kg |
|  | Choline | 1600 mg/kg | 3860 mg/kg |
| Fatty acids | Saturated fats C12:0 or less | ‒ | 1.80% |
|  | Myristic acid 14:0 | 0.03% | 2.6% |
|  | Palmitic acid 16:0 | 0.50% | 7.00% |
|  | Stearic acid 18:0 | 0.14% | 2.40% |
|  | Palmitoleic acid 16:1 | 0.01% | 0.40% |
|  | Oleic acid 18:1 | 1.90% | 5.50% |
|  | Gadoleic acid 20:1 | 0.03% | No data |
|  | Linoleic acid 18:2 n6 | 1.30% | 0.40% |
|  | a linolenic acid 18:3 n3 | 0.30% | 0.20% |
|  | Arachadonic acid 20:4 n6 | 0.01% | Trace |
|  | EPA 20:5 n3 | 0.02% | ‒ |
|  | DHA 22:6 n3 | 0.05% | ‒ |
|  | Total n3 | 0.37% | 0.35% |
|  | Total n6 | 1.31% | 0.41% |
|  | Cholesterol | ‒ | 0.15% |
|  | Total mono unsaturated fats | 2.00% | 6.23% |
|  | Total polyunsaturated fats | 1.77% | 0.77% |
|  | Total saturated fats | 0.74% | 13.99% |

Data as reported by manufacturer; Specialty Feeds, Australia; http://www.specialtyfeeds.com/.
