# Supplementary material for: Paternal Diet-Induced Obesity Retards Early Mouse Embryo Development, Mitochondrial Activity and Pregnancy Health
Source: PLoS One. 2012 Dec 27;7(12):e52304. doi: 10.1371/journal.pone.0052304 (PMC3531483; doi:10.1371/journal.pone.0052304)
Supplement: Table S2 — Composition of handling, IVF and sequential embryo culture media. G-MOPS and Fertilisation media adapted from [22]; G1 and G2 sequential culture media adapted from [23]. (DOCX) [file pone.0052304.s002.docx]

**Table S2. Composition of handling, IVF and sequential embryo culture media.**

| **Component (mM)** | **G-MOPS** | **Fertilisation** | **G1** | **G2** |
| --- | --- | --- | --- | --- |
| NaCl | 90.5 | 100.5 | 90.08 | 90.08 |
| KCl | 5.5 | 5.5 | 5.5 | 5.5 |
| NaH_2_PO_4_•2H_2_O | 0.5 | 0.5 | 0.25 | 0.25 |
| MgSO_4_•7H_2_O | 1.0 | 1.0 | 1.0 | 1.0 |
| NaHCO_3_ | 2.0 | 25.0 | 25.0 | 25.0 |
| CaCl_2_•2H_2_O | 1.8 | 1.8 | 1.8 | 1.8 |
| Glucose | 0.5 | 3.15 | 0.50 | 3.15 |
| Na-Lactate | 10.5 | 10.5 | 10.5 | 5.87 |
| Na-Pyruvate | 0.35 | 0.35 | 0.32 | 0.10 |
| EDTA | ‒ | ‒ | 0.01 | ‒ |
| Alanyl-glutamine | 0.5 | 0.5 | 0.5 | 1.0 |
| Alanine | 0.1 | 0.1 | 0.1 | 0.1 |
| Asparagine | 0.1 | 0.1 | 0.1 | 0.1 |
| Aspartate | 0.1 | 0.1 | 0.1 | 0.1 |
| Glutamate | 0.1 | 0.1 | 0.1 | 0.1 |
| Glycine | 0.1 | 0.1 | 0.1 | 0.1 |
| Proline | 0.1 | 0.1 | 0.1 | 0.1 |
| Serine | 0.1 | 0.1 | 0.1 | 0.1 |
| Taurine | 0.1 | 0.1 | 0.1 | ‒ |
| Arginine | ‒ | ‒ | ‒ | 0.6 |
| Cystine | ‒ | ‒ | ‒ | 0.1 |
| Histidine | ‒ | ‒ | ‒ | 0.2 |
| Isoleucine | ‒ | ‒ | ‒ | 0.4 |
| Leucine | ‒ | ‒ | ‒ | 0.4 |
| Lysine | ‒ | ‒ | ‒ | 0.4 |
| Methionine | ‒ | ‒ | ‒ | 0.1 |
| Phenylalanine | ‒ | ‒ | ‒ | 0.2 |
| Threonine | ‒ | ‒ | ‒ | 0.4 |
| Tryptophan | ‒ | ‒ | ‒ | 0.5 |
| Tyrosine | ‒ | ‒ | ‒ | 0.2 |
| Valine | ‒ | ‒ | ‒ | 0.4 |
| Choline chloride | ‒ | ‒ | ‒ | 0.0072 |
| Folic acid | ‒ | ‒ | ‒ | 0.0023 |
| *i*-Inositol | ‒ | ‒ | ‒ | 0.01 |
| Nicotinamide | ‒ | ‒ | ‒ | 0.0082 |
| Pantothenate | ‒ | ‒ | ‒ | 0.0042 |
| Pyridoxine | ‒ | ‒ | ‒ | 0.0049 |
| Riboflavin | ‒ | ‒ | ‒ | 0.00027 |
| Thiamine | ‒ | ‒ | ‒ | 0.003 |
| MOPS | 23.0 | ‒ | ‒ | ‒ |
| Glutathione | ‒ | 3.25 | ‒ | ‒ |

G-MOPS and Fertilisation media adapted from [22]; G1 and G2 sequential culture media adapted from [23].
